# Almonds (*Prunus Dulcis* Mill. D. A. Webb): A Source of Nutrients and Health-Promoting Compounds

**DOI:** 10.3390/nu12030672

**Published:** 2020-03-01

**Authors:** Davide Barreca, Seyed Mohammad Nabavi, Antoni Sureda, Mahsa Rasekhian, Roberto Raciti, Ana Sanches Silva, Giuseppe Annunziata, Angela Arnone, Gian Carlo Tenore, İpek Süntar, Giuseppina Mandalari

**Affiliations:** 1 Department of Chemical, Biological, Pharmaceutical and Environmental Sciences, University of Messina, 98168 Messina, Italy; rraciti@unime.it (R.R.); gmandalari@unime.it (G.M.); 2 Applied Biotechnology Research Center, Baqiyatallah University of Medical Sciences, Tehran 14359-16471, Iran; nabavi208@gmail.com; 3 Research Group on Community Nutrition and Oxidative Stress (NUCOX), Health Research Institute of the Balearic Islands (IdISBa), and CIBEROBN (Physiopathology of Obesity and Nutrition CB12/03/30038), University of Balearic Islands, Palma de Mallorca, E-07122 Balearic Islands, Spain; tosugo@hotmail.com; 4 Pharmaceutical Sciences Research Center, Health Institute, Kermanshah University of Medical Sciences, Kermanshah 6734667149, Iran; mahsarasekhian@gmail.com; 5 National Institute for Agricultural and Veterinary Research (INIAV), Rua dos Lágidos, Lugar da Madalena, 4485-655 Vila do Conde, Portugal; anateress@gmail.com; 6 Center for Study in Animal Science (CECA), ICETA, University of Oporto, 4051-401 Oporto, Portugal; 7 Department of Pharmacy, University of Naples Federico II, Via Domenico Montesano 49, 80131 Naples, Italy; giuseppe.annunziata@unina.it (G.A.); giancarlo.tenore@unina.it (G.C.T.); 8 Dipartimento di Medicina Clinica e Chirurgia, Unit of Endocrinology, Federico II University Medical School of Naples, Via Sergio Pansini 5, 80131 Naples, Italy; angela.arnone15@gmail.com; 9 Department of Pharmacognosy, Faculty of Pharmacy, Gazi University, 06330 Etiler Ankara, Turkey; ipesin@gazi.edu.tr

**Keywords:** almonds, secondary metabolites, health-promoting properties, almond composition, clinical trials

## Abstract

Almonds (*Prunus dulcis* Miller D. A. Webb (the almond or sweet almond)), from the Rosaceae family, have long been known as a source of essential nutrients; nowadays, they are in demand as a healthy food with increasing popularity for the general population and producers. Studies on the composition and characterization of almond macro- and micronutrients have shown that the nut has many nutritious ingredients such as fatty acids, lipids, amino acids, proteins, carbohydrates, vitamins and minerals, as well as secondary metabolites. However, several factors affect the nutritional quality of almonds, including genetic and environmental factors. Therefore, investigations evaluating the effects of different factors on the quality of almonds were also included. In epidemiological studies, the consumption of almonds has been associated with several therapeutically and protective health benefits. Clinical studies have verified the modulatory effects on serum glucose, lipid and uric acid levels, the regulatory role on body weight, and protective effects against diabetes, obesity, metabolic syndrome and cardiovascular diseases. Moreover, recent researchers have also confirmed the prebiotic potential of almonds. The present review was carried out to emphasize the importance of almonds as a healthy food and source of beneficial constituents for human health, and to assess the factors affecting the quality of the almond kernel. Electronic databases including PubMed, Scopus, Web of Science and SciFinder were used to investigate previously published articles on almonds in terms of components and bioactivity potentials with a particular focus on clinical trials.

## 1. Introduction

The almond (*Prunus dulcis* (Mill.) D. A. Webb) is an important nut native to Central Asia, but today is produced worldwide in hot‒arid Mediterranean climate regions [1]. Nowadays, the USA is the largest almond producer, followed by Spain and Australia. Cultivated almond varieties display a different chemical profile due to genetic and ecological factors, as well as processing conditions. Regular consumption of nuts has been related to healthy effects, especially against cardiometabolic diseases [2,3]. Epidemiological studies and clinical trials have reported positive effects of nuts consumption against a significant number of pathologies such as obesity, hypertension, diabetes mellitus and metabolic syndrome [4,5,6]. In addition, individuals who consume nuts regularly present lower waist circumference and improved metabolic profiles [7]. The almond kernel, which constitutes the edible part, is a seed formed by two large cotyledons covered by a brown skin and protected by an external hull with an intermediate shell [8]. Once maturity is reached, the hull opens and the seed separates easily. Almonds contain lipids (around 50%), proteins (around 25%) and carbohydrates (around 20%), and have a low moisture content and diverse minor bioactive compounds. The beneficial effects of almond consumption are associated with its composition of macro- and micronutrients. Among the compounds with beneficial properties for health, the lipid profile, predominantly monounsaturated fatty acids (MUFA, 60%), followed by polyunsaturated fatty acids (PUFA, 30%), fiber, vitamins, minerals, phytosterols and polyphenols, can be highlighted [9,10]. The great diversity of varieties as well as the forms of cultivation and the climatic characteristics determine the remarkable differences in almonds’ chemical composition [11]. In addition, the form of consumption, mostly raw or roasted, adds additional changes to the composition of almonds. The roasting process induces chemical and microstructural changes, especially altering the lipid composition, favoring its oxidation and modifying antioxidant compounds [12,13]. Moreover, the use of prebiotics to promote gut microbiota modulation towards a health-promoting profile is gaining attention. The fiber and polyphenols content in almonds could be a substrate for microbial fermentation in the gut, contributing to regulation of the microbiota composition [14,15]. Considering the relevance of almonds from an agro-economical and nutritional point of view, the present review addresses the chemical composition of different almond varieties, the bioavailability and metabolism of its components, especially bioactive compounds, and their use as a source of functional foods and prebiotics.

## 2. Food Composition of the Different Varieties

Almonds are tree nuts recognized as a healthy snack and known to be a good source of protein, monosaturated fatty acids, dietary fiber (insoluble/soluble fiber at 4:1), vitamin E, riboflavin and essential minerals (manganese, magnesium, copper and phosphorus) (Table 1, Table 2 and Table 3) [16,17]. Daily intake of 30–50 g is recommended as part of a healthy diet [18]. Generally, in an almond orchard, rows of one variety alternate with one or more rows of other varieties. The selection of the variety depends on the yield, performance of the field in specific regions, resistance to disease, and marketability [17]. The state of California in the United States is one of the main regions of the world producing almonds (around 80% of the world’s almonds). About 30 varieties of almonds are commercialized in California, but only 10 account for most of the production. The most important variety in terms of production and marketing is Nonpareil, due to their excellent tree and nut characteristics [17]. Table 1 summarizes the variability in the nutrient composition of different varieties of almonds. Moreover, it includes the data from two food composition databases, the U.S. Department of Agriculture (USDA) National Nutrient Database for Standard Reference [19] and the Portuguese Food Composition Database (TCA) [20]. National food composition databases, and especially the USDA include the representative values of the almond nutrients found in the national food supply [17]. Some of the differences may be influenced by the water content (3.1–6.5 g/100 g fresh weight) (Table 1), which is related to the nut maturity and harvest and storage conditions [17]. Some of the factors that most affect the variability among almond varieties are genetic, environmental (location, ecological conditions, technical and cultural practices) and analytical [21]. The quality of almonds is generally defined by the water content, lipid content, oil composition and oil ultraviolet absorption coefficients [22]. Therefore, some studies have evaluated the effect of different factors on the kernel quality of almonds. Piscopo et al. [22] studied the effect of harvest time (beginning and end of August) on the fatty acid and mineral contents of different almond varieties. During ripening, an increase in fat and acidic content was observed, and Mas Bovera showed the highest nutritional value. Kazantzis et al. [23] studied the effect of harvest time and storage conditions on two varieties of almonds, Ferragnes and Texas. They concluded that early harvest almonds presented higher moisture, better oil quality and lower sugar content than late harvested almonds. Therefore, they have a higher quality but lower sweetness. Summo et al. [24] also studied the influence of harvest time and cultivar and concluded that harvest time increased the lipid content and decreased the carbohydrates and protein content. Almonds present a similar overall nutrient profile even when comparing different varieties, year of production and growing regions (Table 1, Table 2, Table 3, Table 4 and Table 5). Gama et al. [18] found there were no significant differences in the aluminum, iron, calcium, phosphorus, magnesium, zinc and sodium amount among brands of almond kernels, but the amounts of proteins, potassium, copper, boron, sulfur and manganese were significantly different (Table 1 and Table 2). Moreover, Yada et al. [17] concluded that the difference in mean protein, total lipids, fatty acids and dietary fiber was less than 1.2-fold amongst the seven different almond varieties studied. The highest differences among varieties were found for riboflavin (1.7-fold differences). Almond proteins are recognized to present a high arginine content (Table 5) and good digestibility [16]. Approximately 50% of the almond weight is fat, mostly MUFA, which are associated with the reduction of low-density lipoprotein cholesterol (LDL-c) (Table 1). Therefore, almonds are associated with benefits for cardiovascular health and obesity-related diseases. On the other hand, they may pose a risk for potentially allergic individuals. In a study carried out by Yada et al. [17], the content on β-sitosterol, stigmasterol and campesterol was evaluated in seven almond varieties from the top 10 almond varieties in California and they ranged between 103–206, 1.3–9.8 and 4.1–11.8 mg/100 g, respectively. Summo et al. [24] also studied the antioxidant activity and total phenolic compounds of 10 almond cultivars and found a great variability influenced by the genotype. Also, late harvest increased antioxidant activity, suggesting that antioxidant compounds develop in the late stage of ripening.

## 3. Main Almond Polyphenols

**Figure 1:**
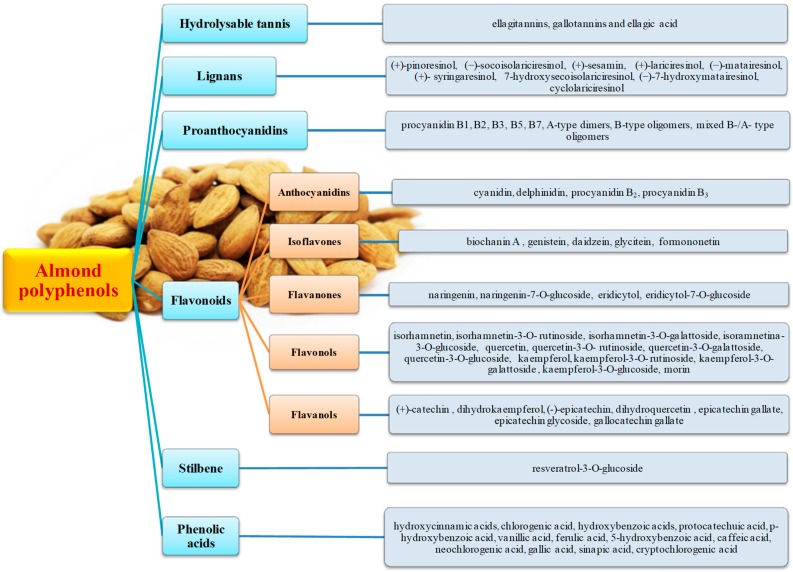
Main polyphenols present in whole almonds.

Polyphenols are concentrated at the lipid interface to the environment, where they are responsible for the almond quality, contributing to the properties and increasing the shelf life. Based mainly on high performance liquid chromatography-diode array- mass spectrometry detection (HPLC-DAD-MS) and gas chromatography–mass spectrometry (GC-MS) separation techniques, numerous polyphenols (~312 mg/100 g of almond) have been identified in whole almonds, but also in almond blanch water, almond skins and almond hulls [29,30,31,32]. Among polyphenols, the most abundant were hydrolysable tannins, proanthocyanidins and flavonoids (Figure 1), independently of the almond’s nature (roasted or raw), the cultivar, the cultivation techniques and the environmental factors, ranging from 61 to 162 mg/100 g of almonds. Phenolic acids, lignans, isoflavones and stilbenes (Figure 1) were identified in lesser, but always significant, amounts ranging from 0.7 to 5.5 mg/100 g of almonds. Almond proanthocyanidins are mainly characterized by B-type interflavan bonding (with carbon–carbon bonds at C4→C6 or C4→C8), based on regular repetition of (−)-epicatechin and (+)-catechin, and, in minor amounts, of (−)-epiafzelechin [29]. These basic elements can be further modified by galloylation or polygalloylation [33,34]. A-type proanthocyanidins have been reported, but they have not been well characterized [29]. Almonds are rich in proanthocyanidin polymers, but B-type (procyanidin B1, B2, B3, B5, B7) and A-type dimers, B-type oligomers, and mixed B-/A-type oligomers have also been identified by normal-phase high performance liquid chromatography (HPLC) analysis, high performance liquid chromatography-mass spectrometry (HPLC-MS) methods and matrix-assisted laser desorption/ionization (MALDI) analysis [33,34,35,36,37]. Among the hydrolysable tannins, ellagitannins, gallotannins and ellagic acid have been separated and identified in almonds, with contents ranging from 53–57, 20–34 to ~0.51 mg/100 g, respectively [38,39].

Different classes of flavonoids have been reported in almonds or their coproducts, including flavonols, anthocyanidins (cyanidin), flavanones and flavan-3-ols. Among the flavan-3-ols, (+)-catechin, dihydrokaempferol, and (-)-epicatechin were the most abundant molecules, ranging from 2 to 40 mg/100 g of almond, but, although in minor amounts, dihydroquercetin, gallocatechin gallate, epicatechin gallate and epicatechin glycoside have also been reported [29,40]. Flavonols are, by far, the most abundant flavonoid class in almonds (ranging from 87 to 135 mg/100 g) and include kaempferol, quercetin, isorhamnetin and their 3-*O*-glucosides, rutinosides and galactosides and morin [41,42]. Isorhamnetin and its derivatives are the most abundant compounds, followed by kaempferol and quercetin derivates. Naringenin and its 7-*O*-glucoside derivatives are the main flavanones identified in whole almonds (~3 mg/100 g), while eridicytol-7-*O*-glucoside is present in almond skin and blanch water. Several different phenolic acids and aldehydes (ranging from 5 to 12 mg/100 g) have been reported in whole almonds, such as hydroxycinnamic acids, chlorogenic acid, hydroxybenzoic acids, protocatechuic acid, *p*-hydroxybenzoic acid, vanillic acid, ferulic acid, 5-hydroxybenzoic acid, caffeic acid, neochlorogenic acid, sinapic acid and cryptochlorogenic acid [43,44]. Limited information is available on isoflavones, lignans and stilbenes, with the identification, in most cases, of aglycone structures, after chemical or enzymatic hydrolysis, but it is possible that these structures are present in the glycosylated forms as well as involved in the formation of more complex structures in the natural matrix. Although some isoflavones have been quantified in whole almonds (~40 μg/100 g), several derivates have been identified by high performance liquid chromatography-mass spectrometry (HPLC-MS) and gas chromatography–mass spectrometry GC-MS, including biochanin A (by far the most abundant derivative ~25 μg/100 g), genistein, daidzein, glycitein and formononetin [45]. Resveratrol-3-*O*-glucoside is the main stilbene identified and quantified, while, after hydrolysis, a combination of GC-MS and HPLC-MS/MS analysis has led to the identification of the following lignans: (+)-pinoresinol, (−)-socoisolariciresinol, (+)-sesamin, (+)-lariciresinol, (−)-matairesinol, (+)- syringaresinol, 7-hydroxysecoisolariciresinol, (−)-7-hydroxymatairesinol and cyclolariciresinol in a total amount of ~670 μg/100 g [45,46].

## 4. Bioavailability and Metabolism of Almond Components

**Figure 2:**
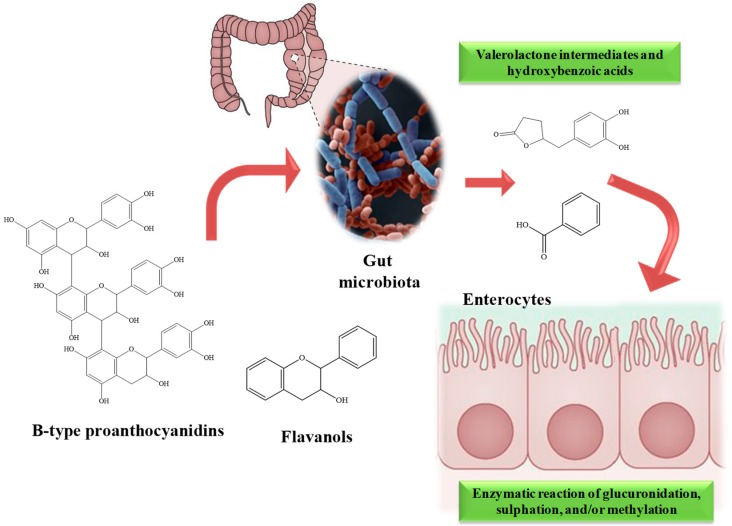
Schematic representation of metabolization processes of flavanols and B-type proanthocyanidins, catalyzed first by gut microbes with the formation of small phenolic compounds and inside the entherocytes by Phase I and II enzymes.

Almonds contain several compounds with remarkable functions in the human body. They are rich sources of carbohydrates (mainly dietary fiber), fatty acids, proteins and amino acids, as well as vitamins, minerals and secondary metabolites. Mastication has a significant impact on the following phases of digestion, although other factors can also influence the process. Experiments performed in vivo on pigs showed no significant statistical differences in the plasma glucose or lipid amount or in particle sizes and rheological behavior during gastric digestion between raw and roasted almonds [47,48], although the authors noticed a more rapid gastric emptying of protein in pigs for raw than roasted almonds due the process of protein segregation. Moreover, recently, Grundy et al. [49] detected negligible differences, during the duodenal phase, in the release of lipid in the gastric compartment and during the time course of lipid digestion [50] between the masticated bolus of roasted almonds and raw almonds. In the first phase of digestion, the particle size plays a substantial role in the release of the components for their absorption and metabolization. In fact, from proteins to carbohydrates and lipids, mastication itself has an effect on nutrient digestion and absorption in the gut, on weight management, hormone release and satiety. Mandalari et al. [51] provided a possible mechanism for incomplete nutrient absorption in the gut, due to the presence of almond cell walls that prevent lipid release from intact cells, Moreover, recent researchers have shown the fundamental role of almond cell walls in the regulation of almond components, postprandial lipemia and glycemia and of consequences in the attenuation, for instance, of cardiovascular disease risk. Almonds are rich sources of polyphenols, which are metabolized remarkably after their ingestion, and the bioavailability of these compounds significantly influences the health-promoting properties of this matrix as well as of its co-products and derivatives. In fact, the metabolization of polyphenols may result in the production of different classes of metabolites that, often, may have more interesting biological activities than their dietary natural precursor. Almond tannins, for instance, are mainly utilized and transformed by the gut microbiota, producing valerolactone intermediates and hydroxybenzoic acids after the metabolization of proanthocyanidins (Figure 2). Following this metabolization, the products are released from the gut microbiota, absorbed and submitted to enzymatic transformation to produce hydroxyl, glucuronic, sulfate and/or methylated derivatives. Studies related to the bioavailability of almond polyphenols are also available in human population after the consumption of whole nuts or its crude extracts. In a first study, Urpi-Sarda et al. [39] analyzed and characterized the polyphenols and their metabolites present in the plasma and urine of healthy human subjects who consumed almond skin polyphenols [39]. Bioproducts of phase II and III enzymes (O-methyl glucuronide, sulfate, glucuronide and O-methyl sulfate derivatives) of naringenin, (epi)catechin and isorhamnetin were identified in plasma and urine samples in the nanomolar range, along with the glucuronide and sulfate forms of 5-(dihydroxyphenyl)-γ-valerolactone and 5-(hydroxymethoxyphenyl)-γ-valerolactone (the main bioproducts of microbial flavanols’ metabolization). Moreover, several other metabolites, present in minor amounts, were found in the urine samples, always deriving from the microbial metabolization of polyphenols such as hydroxycinnamic, hydroxyphenylpropionic, hydroxybenzoic, hydroxyphenylacetic, and hydroxyhippuric acids (in their hydroxyphenylvalerolactone form). Bartolomé et al. [52], using a combination of analytical techniques (LC/ESI-MS, LC-DAD/fluorescence and MALDI-TOF-MS), identified O-methyl glucuronide, O-methyl sulfate, sulfate and glucuronide derivatives of (epi)catechin, the glucuronide conjugates of isorhamnetin and naringenin, and sulfate conjugates of isorhamnetin, together with conjugates of hydroxyphenylvalerolactones and several products of microbial metabolization products such as hydroxyphenylacetic, hydroxyphenylpropionic, hydroxyhippuric, hydroxycinnamic and hydroxybenzoic acids in plasma and urine samples. Garrido et al. [53] performed a subsequent single-blind, placebo-controlled, and randomized trial study on 16 healthy volunteers (nine men and seven women) with the aim of analyzing the changes in the urinary excretion of almond skin polyphenols and their metabolized products after different time intervals (0–2, 2–6, 6–10 and 10–24 h) from intake. The control group ingested microcrystalline cellulose, while the test group ingested 884 mg of encapsulated flavan-3-ols, flavonols and flavanones’ almond skin polyphenols. The maximum urinary excretion of naringenin and (epi)catechin conjugates was reached between 2 and 6 h after consumption, while conjugated metabolites of isorhamnetin and hydroxyphenylvalerolactones reached the maximum between 10 and 24 h.

## 5. Health-Promoting Properties: Evidence from Intervention Clinical Trials

The use of almonds as a health-promoting food dates back a long time; indeed, the ancient Greeks, Persians, Chinese and Indians habitually consumed them for medical purposes in their traditions [54]. In the last 20 years, the consumption of almonds has risen significantly [55], suggesting that both in the general population and for producers the perception of these nuts has evolved from a convenient snack to an essential food for maintaining human health [56]. In a large body of studies, indeed, the consumption of almonds has been associated with various health benefits, including the modulation of serum lipid [57,58,59,60] and glucose levels [61,62,63,64,65], the regulation of body weight [66], and protection from several diseases, such as diabetes [67,68], obesity [69,70,71] and cardiovascular diseases [64,72,73,74,75,76,77]. In the present section, we summarize the most relevant evidence, derived from clinical trials carried out on humans, demonstrating the beneficial health effects of almond consumption. The studies have been grouped on the basis of their main outcomes. As shown in Table 6, the studies significantly differ in terms of population type and number, amount and source of intervention and duration. However, the most significant results are generally in agreement, providing solid evidence so physicians and nutritionists can feel confident in encouraging the use of almonds in the management of several borderline conditions.

### 5.1. Lipidemic Control

One of the predominant beneficial effects of regular almond consumption is the control of blood lipid levels. A randomized controlled trial meta-analysis reported the association between almond consumption and the reduction of both total cholesterol (TC) and LDL-c; however, no significant effects have been observed in terms of high-density lipoprotein cholesterol (HDL-c), triglycerides (TG) or LDL-c/HDL-c ratio [82]. Similar results were provided by a study demonstrating that, in subjects with mild hypercholesterolemia, supplementation with 20 g almonds daily for six weeks significantly improved the lipid profile. In particular, a reduction in TC (−8.1 ± 2.4%, *p* = 0.007), LDL-c (−9.4 ± 2.4%, *p* = 0.005) and non-HDL-c (−8.1 ± 3.0%, *p* = 0.013) serum levels was observed, compared to the placebo (corn starch capsules); however, the results for HDL-c and TG were not significant (−4.7 ± 2.3%, *p* = 0.06 and −10.4 ± 7.0%, *p* = 0.08, respectively) [58]. Similar results have been reported for hyperlipidemic subjects supplemented with 10 mL almond oil twice daily for four weeks, demonstrating a significant reduction in TC (from 224.95 ± 33.59 to 208.69 ± 28.89 mg/dL, *p* = 0.001) and LDL-c serum levels (from 138.76 ± 20.60 to 131.05 ± 17.89 mg/dL). Furthermore, no statistically significant effects have been observed on TG and HDL-c [60]. However, in addition to reduction of TC and LDL-c levels, improvements in TG and HDL-c levels after almond supplementation have been observed in normolipidemic subjects [57]. Twenty-two (22) healthy men and women were randomized into two intervention groups consisting of supplementation with whole almonds or almond oil; supplementation replaced 50% of the usual daily intake of dietary fat and was followed for six weeks. After the treatment period, researchers observed significantly reduced levels of TC, TG and LDL-c and increased HDL-c in both intervention groups compared to the baseline values (*p* < 0.05 for all parameters) [57], suggesting that almonds are useful for ameliorating the lipid profile, independently of the dietary source. According to the authors, the TG-lowering and HDL-c-increasing effects of almond supplementation might be due, at least in part, to their peculiar composition, characterized by high levels of unsaturated fatty acids, including MUFA and PUFA, whose effects in terms of ameliorating lipid profile are well established. Various authors agree on the beneficial role of the almond lipid fraction, which, in addition to other components, such as sterols and flavonoids, may be considered the main factor explaining the lipid-lowering effect of almonds [83]. Moreover, the authors also speculated further mechanisms involving improvements in both body weight and body composition. Although some studies did not demonstrate significant changes in body composition after supplementation with almonds (as will be discussed below), in healthy subjects long-term supplementation (20 weeks) with 56 g almonds daily resulted in improvements to the lipid profile (reductions in TC, LDL-c, non-HDL-c and TG serum levels) and decreased fat mass and waist-to-hip ratio [59], suggesting that, at least in this class of subjects, nut consumption may positively affect the body’s fat storage, pointing to a further possible mechanism of the lipid-ameliorating effect.

### 5.2. Glycemic Control

In addition to lipidemic control, various studies focused on the role of almond supplementation in modulating glucose homeostasis [61,62,63,64,65,66,67,68,69,70,71,72,73,74,75]. As shown in Table 6, the studies are rather different in terms of their study populations, intervention, duration and outcomes. Indeed, interventions have been carried out on healthy subjects [61,63], diabetics [64,65] or both [62]. Furthermore, two studies investigated the acute effect of almond ingestion on postprandial glucose homeostasis, using standardized test meals [61,62]. In 2006, Jenkins and colleagues [61] demonstrated the effect of a meal containing almonds on glucose and insulin response and oxidative stress. Fifteen (15) 12 h-fasting healthy subjects followed five different sessions consisting of four different meals: white bread (as the control, on two occasions), 60 g almonds with white bread, parboiled rice and mashed potatoes. Each meal contained 50 g of carbohydrates and was balanced for fat and protein (except for the white bread). The protocol was planned with a one-week wash-out period between each session. Among the main results, the authors observed that the almond-containing meal positively affected satiety (the incremental response area was greater than the control after 2 h and 4 h, *p* = 0.047 and 0.011, respectively), postprandial glycaemia (peak heights: almonds = 106 ± 4 mg/dL, rice = 104 ± 2 mg/dL, potatoes = 119 ± 4 mg/dL, bread = 124 ± 4; *p* < 0.001 for all), insulinemia (peak heights: almonds = 32.25 ± 3.45 µIU/mL, rice = 32.42 ± 4.03 µIU/mL, potatoes = 55.87 ± 4.32 µIU/mL, bread = 46.22 ± 5.18 µIU/mL; *p* < 0.001 almonds vs. potato and ≤0.042 almonds vs. bread) and oxidative stress, assessed through an evaluation of protein thiol concentrations (changes in serum protein thiol concentrations: almonds = 15 ± 14 mmol/L, combination of data from the other three meals = -10 ± 8 mmol/L; *p* = 0.021) [61]. Overall, these data suggest that the addition of almonds to a meal significantly improves both the glycemic and insulinemic control (decreasing the meal glycemic index, probably due to their fat, protein and fiber content) and reduces oxidative stress-induced protein damage, which commonly occurs as a consequence of the prolonged hyperglycemia; this last effect may also be due to the high content of antioxidant compounds, mainly polyphenols. A similar study was conducted by Cohen and Johnston [62] investigating the effect of almond consumption during a meal in both healthy subjects and patients with type 2 diabetes mellitus (T2DM). Study participants were randomized into two groups, consuming a standardized meal with or without almonds (28 g). Both meals were isocaloric, with equal amounts of carbohydrates and fats. After a one-week wash-out period, subjects in one group moved to the other, following a crossover study design. Interestingly, researchers observed that almond consumption significantly reduced the postprandial glycaemia only in T2DM subjects (−30%, *p* = 0.043); on the other hand, serum levels of insulin and glucagon-like peptide-1 (GLP-1) were not significantly impacted in either group. In the same study, the authors also carried out a small chronic study on T2DM patients randomized into two groups: almond supplementation (1 oz.) and cheese (two sticks); both intervention groups consumed this diet five days per week for 12 weeks. After the intervention period, significantly reduced levels of glycosylated hemoglobin (HbA1c) were found in the almond group [62]. According to the authors, the observed hypoglycemic effect of almonds might be due to the slowing of gastric emptying (as a consequence of the fat and protein contained in the nuts), but also to the presence of polyphenols, mainly flavonoids, that help control glucose blood levels [62,84], inhibiting amylase [62]. Interestingly, flavonoids are effective ay inhibiting the activity of salivary amylase, which represents a remarkable target for glycemic control in diabetics since in this class of patients the activity of pancreatic amylase is strongly reduced (28% to 35%) [85,86]. Further studies investigated the effect of almond consumption in chronic diseases. It has been demonstrated that in healthy adults eight-week supplementation with 56.7 g almonds significantly reduced fasting glucose, although, similarly to the study of Cohen and Johnston [62], insulin and glucagon-like peptide 1 (GLP-1) levels were not impacted; however, researchers observed increased sensitivity to insulin, evaluated by both the insulin resistance index and the Matsuda index as 34% lower and 82% higher, respectively, than the control [63]. In T2DM patients, long-term supplementation with almonds (four and 24 weeks) resulted in significantly reduced HbA1c (from 7.7 ± 1.2 to 7.3 ± 1.1%, *p* = 0.04) [47], fasting glucose (4.1%, *p* = 0.023), fasting insulin (0.8%, *p* = 0.018) and homeostatic model analysis for insulin resistance (HOMA-IR) (9.2%, *p* = 0.004) [65].

### 5.3. Obesity

The two studies reported in Table 6, demonstrating the role of almond consumption in overweight [70] and obese subjects [70,71], present quite different results. A 12-week clinical trial was carried out with 86 healthy subjects with a body mass index (BMI) that ranged from 25 to 40 kg/m^2^ (thus classified as overweight or obese), who were randomized into two diet intervention groups: an almond-enriched hypocaloric diet (AED, 15% of total kcal from almonds) and a nut-free hypocaloric diet (NFD). Each diet provided a daily 500-kcal deficit, with the energy requirement estimated by predictive equations. After the intervention period, although subjects in both groups lost body weight, in those that followed the AED significantly higher reductions in total and truncal fat mass were observed, as well as an increase in the total and truncal fat-free mass (*p* < 0.05). According to the authors, these results might be mainly due to the high UFA content in almonds, which have high fat oxidation rates that contribute to reducing visceral fat [70]. In 2012, Foster and co-workers [71] conducted a very long-term diet intervention trial (18 months) on obese subjects (baseline BMI: 34.0 ± 3.6 kg/m^2^), randomized into AED (28 g almonds daily) or NFD diet intervention groups. Although there were positive results after the 18-month intervention period, the researchers observed no statistically significant differences between the two groups in terms of weight loss, body composition or blood pressure; however, significantly decreased levels of TG and TC were observed in the AED group at six months but not at the end of the study [71].

### 5.4. Cardiovascular Risk

The role of impaired glucose and/or lipid homeostasis and obesity in contributing to cardiovascular risk has been well established, either as single or combined risk factors. It appears clear, therefore, that therapeutic strategies, as well as dietary approaches aimed to counteract these metabolic alterations, result in reduced cardiovascular risk. According to the aforementioned evidence, this might be enough to recommend the regular consumption of almonds to achieve a reduction in cardiovascular risk in susceptible subjects. However, various studies directly investigated the role of almond consumption in various factors related to cardiovascular diseases, including the glucose and lipid profile [64,72,73,74,75,76,77], body fat [64,72], inflammation-related markers [64,73] and vascular outcomes [73]. However, the reported studies have differences in terms of population, intervention, duration and results, providing no weighted evidence for a general conclusion on the positive role of almond consumption in the management of cardiovascular diseases. In general, as reported in Table 6, all the studies demonstrate significant improvements in (i) blood lipids, in terms of reduced levels of TC, TG [64,74], LDL-c [64,72,75,77], non-HDL-c [72], ApoB 100 [64], lipoprotein A, oxidized LDL-c [75]; (ii) glucose homeostasis, in terms of reduced HbA1c [64], insulin, HOMA-IR and HOMA for beta-cell function (HOMA-β) [77]; (iii) body composition, in terms of reduced waist circumference, waist-to-height ratio [64] and abdominal and leg fat [72]; and (iv) inflammatory status [64].

In 2015, Chen and colleagues [73] conducted a study on 45 patients with coronary artery disease following a six-week diet with 85 g almonds daily. After the intervention period, the researchers observed no significant improvements in vascular outcomes, evaluated by measuring the flow-mediated dilation, one of the most accredited techniques for monitoring the endothelial function; similarly, no significant improvements were observed in serum parameters, including lipids, C reactive protein (CRP), tumor-necrosis factor (TNF)-α and E-selectin. However, they demonstrated a slightly significant reduction of vascular cell adhesion molecule-1 (−5.3%, *p* = 0.064) and a nonsignificant increase in urinary nitric oxide (+17.5%, *p* = 0.112), suggesting that almonds might modestly contribute to improving the endothelial function [73]. Interestingly, two randomized, crossover studies from the same authors directly investigated the effect of almond consumption on the risk of coronary heart disease (CHD) using the Framingham’s 10-year risk score [75,76]. Both studies were carried out on 27 hyperlipidemic men and postmenopausal women randomized into three one-month diet intervention groups: control (muffin, 147 ± 6 g/day), full-dose almonds (73 ± 3 g/day) and half-dose almonds (37 ± 2 g/die)+half-dose muffin (75 ± 3 g/day); during the intervention period, all subjects were instructed to follow their noncontrolled low-fat diet. After the intervention period, significant improvements in lipid profile were observed in both the almond intervention groups, following a dose-dependent manner [75,76]; in particular, it was estimated that every ~7 g almonds daily are able to reduce LDL-c by ~1%, resulting in a 2% reduction of CHD risk (CHD risk: −9.2 ± 3.5%, *p* = 0.015, full-dose almond group) [75]. Notably, an association was observed between the consumption of almonds and the reduction of the Framingham 10-year CHD risk score (*R* = −0.247, *p* = 0.026), which translates into a 3.5% reduction of CHD risk for every 30 g increase in almond intake [76]. Nevertheless, although they should be interpreted with caution, the data presented herein might allow for considering almonds as a useful dietary approach in the long-term prevention of cardiovascular risk.

### 5.5. Inflammation and Oxidative Stress

The anti-inflammatory potential of almond consumption has been investigated through a randomized, crossover study [78] involving 25 healthy subjects, randomized into three–four week diet interventions: control cholesterol-lowering nut-free diet, low-almond diet (10% of total energy) and high-almond diet (20% of total energy). After the intervention period, E-selectin levels were significantly lower in the high-almond group compared to the placebo (−7.8%); interestingly, it was estimated that for every 1% increase in energy replaced with almonds, E-selectin decreased by 0.18 µg/L. On the other hand, both of the almond diets significantly reduced serum levels of C-reactive protein (CRP). According to the authors, the anti-inflammatory effects might be mainly attributed to the high MUFA content, which has been considered responsible for the decreased levels of E-selectin and CRP. Additionally, further almond components, including magnesium, arginine and phytochemicals, may also contribute to reducing the levels of inflammatory mediators [78]. The antioxidant activity of almonds has been evaluated in two studies on habitual smokers [79,80]. A pilot study was conducted on 30 young subjects habitual smoking 10–20 cigarettes daily, and with at least a five-year smoking history. Subjects were randomized into three groups (*n* = 10 per group): control (no almonds), 84 g and 168 g almond supplementation daily for four weeks. After the treatment period, the percentage of tail DNA was reduced in both the almond-supplemented groups, with statistically significant differences in the higher-dose group (*p* < 0.05 compared to the control group); in addition, in both the almond-supplemented groups significantly reduced levels of 8-hydroxy-2′-deoxyguanosine (8-OH-dG) and malondialdehyde (MDA) (*p* < 0.05 for all, compared to the control group) were found. However, no significant effects were observed for superoxide dismutase (SOD) and glutathione peroxidase (GSH-Px) [79]. Subsequently, the same researchers, starting from these preliminary results, carried out another similar study on a larger cohort with the same inclusion criteria. Also, in this case, after the intervention period, DNA damage, MDA and 8-OH-dG were significantly reduced (23%, 34% and 28%, respectively); however, significantly increased levels of SOD and GSH-Px were also observed. According to the authors, this discrepancy of the results from the pilot study can be explained by the higher sample size in the subsequent study [80]. As the authors claimed, these beneficial effects should be attributed to the high content of antioxidant compounds in almonds, mainly present in the skin [79]. Overall, these results suggest that almond consumption may contribute to protection against oxidative stress and related consequences among smokers.

### 5.6. Serum Uric Acid

A large, randomized, nut-free diet-controlled study demonstrated the uricemia-lowering effect of 12 weeks of regular consumption of almonds (10 g daily) in 150 coronary artery disease patients. After the intervention period, at week 6 and week 12 significantly reduced serum levels of uric acid were registered in both men (−15% and −17%, respectively; *p* < 0.05 for all, compared to nut-free diet group) and women (−12% and −16%, respectively; *p* < 0.05 for all, compared to nut-free diet group) [81].

### 5.7. Secondary Metabolites of Almonds as a Functional Food and Prebiotics

Several secondary metabolites have been identified in almonds, mainly represented by the polyphenols found in the skin. Although the polyphenol amount and antioxidant activity of almonds from California is a function of the cultivar and the harvest year [30], the mean and 25% to 75% percentile contents reported in the literature were (per 100 g almonds): 162 mg (67.1 to 257 mg) proanthocyanins, 82.1 mg (72.9 to 91.5 mg) hydrolysable tannins, 61.2 mg (13.0 to 93.8 mg) flavonoids (non-isoflavone), 5.5 mg (5.2 to 12 mg) aldehydes and phenolic acids, and 0.7 mg (0.5 to 0.9 mg) stilbenes, isoflavones, and lignans [31]. The extraction of the seed with solvent releases additional phenolic acids, proanthocyanins and phenols. The influence of roasting, pasteurization and storage on the almond skins’ polyphenol content has been widely investigated: Bolling et al. [32] reported that roasting led to a decrease in total phenols and ferric reducing antioxidant power but did not affect the amounts of flavonoids and phenolic acids. On the contrary, Garrido et al. [87] found that roasted almonds had the highest antioxidant activity in terms of ORAC values. It is well known that industrial blanching for the removal of the skin consistently reduces almonds’ polyphenol content, since most of the water-soluble compounds end up in the water and the blanched skin [44,88]. A recent study has demonstrated that polyphenols in almond skins after blanching were able to modulate plasma biomarkers of oxidative stress, including glutathione status, glutathione peroxidase activity and resistance of LDL to oxidation in healthy humans [89]. The polyphenols retained in the blanched water, which is currently considered a byproduct of the almond processing industry, could be further utilized for their antimicrobial and antiviral properties [90,91]. There are just a few scientific works that have analyzed the contribution of polyphenols to the health-promoting activity of almonds; the first preclinical studies of polyphenol-rich almond skin or almond extracts, for instance, showed the direct involvement of these components in biological activities such as antiviral activity, antioxidant, detoxicant, anti-inflammatory, anti-platelet aggregation activities and UV protection [31,92]. Therefore, almond skins have a unique polyphenol profile contributing to both food quality and health-promoting functions. The possibility of using almond bioactives as additives in food could be explored: the need for natural food additives as a replacement of synthetic compounds has notably increased over the last decade [92]. Almond polyphenols have antioxidant, antimicrobial and antiviral properties, appealing as a natural food preservative. For example, the addition of almond skin in powder form reduced lipid oxidation in raw chicken breasts under refrigeration and freezing conditions [93]. Almond skin powder also reduced oxidation and increased the shelf life of ground beef [94] and can be used as a preservative in products containing nuts [95]. Further development and optimization could promote the use of almond skin polyphenols in the food industry. A number of studies have indicated that the consumption of almonds and almond skins may lead to an improvement in the intestinal microbiota composition, inducing the growth and/or activity of beneficial bacteria [96,97,98]. In one study performed by Mandalari et al. [16], finely ground almonds and defatted finely ground almonds were utilized in a combined model of the gastrointestinal tract (in vitro gastric and duodenal digestion), followed by microbial metabolization of the resulting fractions to analyze their influences and activities on the composition and metabolic potential of gut bacteria populations. Finely ground almonds significantly stimulate the growth of bifidobacteria and *Eubacterium rectale* populations, whereas there were no significant differences in response to defatted finely ground almonds. Liu et al. [96] have analyzed the prebiotic effects, on healthy humans, of almond and almond skin intake. The analysis of fecal samples over six weeks after intervention showed a significant increase in *Bifidobacterium* spp. and *Lactobacillus* spp. populations, whereas *Escherichia coli* remained stable and *Clostridum perfringens* growth was significantly repressed. The gut microbiota composition was analyzed after the consumption of almonds (0, 1.5 or 3 servings/day for 18 days): no increase in lactobacilli and bifidobacteria was detected [97]. In a four-week animal trial, consumption of raw and roasted almonds led to an increase of *Bifidobacterium spp*. and *Lactobacillus spp*. numbers, decreasing the growth of *Enterococcus spp*. [14]. Raw almonds led to an increase in bifidobacteria numbers, associated with higher β-galactosidase activity and lower β-glucuronidase and azoreductase activity compared with roasted almonds. Fiber and other components in almonds and almond skins have potential prebiotic properties. Mandalari et al. [98] investigated the prebiotic effect of natural (NS) and blanched (BS) almond skins in vitro. Both NS and BS significantly increased the bifidobacteria and *Clostridium coccoides/Eubacterium rectale* numbers. This study demonstrated that the presence of polyphenols in almond skins did not affect bacterial fermentation, and that almond skins, which are by-products of industrial blanching, could potentially be used as prebiotics.

## 6. Conclusions 

In the present review, we have discussed the nutritional value, biological activities and phytochemical profile of almonds, a popular and important medicinal and dietary nut with a long history of use. The kernels are rich in fat (MUFA and PUFA), carbohydrates (fiber, etc.), proteins, vitamins (vitamin E, vitamin B, etc.), minerals (copper, calcium, magnesium, etc.) and diverse bioactive compounds (phytosterols, polyphenols, etc.) and are used as a natural antioxidant and anti-inflammatory. The abundant micronutrient polyphenols play an important role in protection from chronic degenerative diseases. Indeed, frequent consumption of almonds has been associated with reduced risk of various diseases, including obesity, hypertension, diabetes mellitus and metabolic syndrome, owing to their LDL-c reducing ability. These activities were attributed to the lipid fraction and polyphenolic compounds of almonds. However, the health benefits of these components depend on their bioavailability and ingested amount. It was also reported that the consumption of almonds and their skin may improve the intestinal microbiota profile and lead to health benefits. A lot of evidence suggests the health-promoting properties of almonds, though several aspects remain unrevealed, since the influence of microstructural components on the release of phytonutrients from this complex matrix and the potential of bioactive compounds to activate intracellular signal cascade and modulate complex processes inside the organism are still poorly understood.

## Figures and Tables

**Table 1:** Nutritional composition (g/100 g) of different varieties of almonds (*Prunus dulcis* Mill. D. A. Webb).

	Variety	Unknown	Unknown	Butte	Carmel		Desmajo Largueta	Ferragnés	Filippo Ceo	Francoli	Fritz	Genco	Johnston Prolific	Marcona	Mission		Monterey	Nonpareil		Sonora	Texas	Thompson	Tuono
Nutrients																							
Water		4.4	4.9	4.7	3.1	4.1	5.7	6.5	5.4	5.7	4.6	6.5	5.4	6.1	4.3	4.6	3.9	4.0	3.9	4.1	6.5	5.3	5.3
Protein		21.2	21.6	20.5	20.6	20.2	19.6	18.1	14.1	20.5	22.5	21.5	20.2	22.1	23.3	20.9	21.3	21.0	20.2	22.4	20.1	21.2	20.5
Total lipids		49.9	56.0	50.0	47.5	50.1	50.6	50.1	56.2	44.3	48.4	42.4	47.0	52.7	44.1	49.6	49.4	43.3	49.6	50.2	48.8	48.0	47.3
SFA				4.1		3.9					3.4					3.7	3.7		3.8	3.9			
MUFA				29.4		29.7					30.5					31.6	32.3		31.3	31.4			
PUFA				13.9		13.8					12.0					11.6	11.2		11.7	12.4			
Ash		3.0	3.0	2.8	3.7	2.9	3.2	2.9	2.5	3.5	2.9	3.0	2.6	3.4	4.6	3.0	3.0	4.5	2.9	3.0	2.9	3.1	3.0
Carbohydrate		21.6	19.2		25.0		20.9	22.4	21.8	26.1		26.6	24.8	15.7	23.6			27.0			21.7	22.4	23.9
Fiber		12.5	12.0	12.2		12.5					11.0					13.5	11.8		12.9	11.8			
Sugars		4.4			5.4										6.2			7.5					
References		[19]	[20]	[17]	[25]	[17]	[24]	[24]	[24]	[24]	[17]	[24]	[24]	[24]	[25]	[17]	[17]	[25]	[17]	[17]	[24]	[24]	[24]

**Table 2:** Mineral composition (mg/100 g) of different varieties of almonds (*Prunus dulcis* Mill. D. A. Webb).

	Variety	Unknown	Unknown	Mixture	Butte	Carmel	Falsa Barese	Ferragnés	Fritz	Genco	Glorieta	Lauranne	Mas Bovera	Mission	Monterey	Nonpareil/Monterey	Nonpareil	Pepparuda	Sonora	Stelliette	Supernova	Trianella	Tuono
Nutrients																							
Aluminum (Al)				0.39																			
Boron (B)				1.85																			
Calcium (Ca)		269	270	200	288	279	137–142	128–138	290	138	90–102	166–176.5	103–129	330	252	172–252	261	118–131	234	155–176	113–127	106–125	106–127
Iron (Fe)		3.7	4	3.22	3.27	3.27	3.13–4.45	3.42–4.48	3.63	1.82–4.29	4.36–6.10	4.45–4.97	1.56–4.73	3.34	3.58		3.47	2.84–3.45	3.84	4.09–4.27	5.54–6.49	2.80–5.38	2.71–5.11
Magnesium (Mg)		270	260	300	263	262	235–254	226–233	260	254–235	245–249	154–246	256–276	272	278	121–145	275	223–246	256	241–249	218–234	226–241	222.5–225.5
Phosphorus (P)		481	410	500	463	462			466					512	524	191–258	455		526				
Potassium (K)		733	860	600	664	679	690–726	661–695	664	572–674	694–727	652–737	733–786	724	766	1550–2370	762	729–745	773	699–794	659–672	525–727	676–741
Sodium (Na)		1	6	3.54																			
Sulphur (S)				200																			
Zinc (Zn)		3.1	3.1	21.3	2.98	2.77	2.83–5.51	3.71–4.89	2.82	3.95–4.69	4.66–5.55	4.45–5.93	3.88–5.46	2.76	2.79		3.23	1.08–4.99	3.8	4.37–5.53	3.25–5.30	4.38–5.37	4.3–5.1
Copper (Cu)		1		0.89	0.92	1.09	1.98–3.38	2.14–3.80	0.85	1.83–3.90	1.93–3.85	2.18–3.76	1.68–3.82	0.72	0.94		1.05	2.27–8.61	0.9	1.76–3.72	2.16–2.89	2.06–3.74	2.04–3.58
Manganese (Mn)		2.2		2.56	2	2.14	1.01–1.27	1.04–1.46	2.08	1.16–1.29	1.11–1.12	1.22–1.44	1.39	2.2	2.12		2.21	0.89–0.95	3.04	1.21–1.32	0.93–1.26	1.16–1.20	1.21–1.38
Selenium (Se)		4.1 × 10^-3^																					
Units		mg/ 100 g FW	mg/ 100 g FW	mg/ 100 g	mg/ 100 g FW	mg/ 100 g FW	mg/ 100 g DW	mg/ 100 g DW	mg/ 100 g FW	mg/ 100 g DW	mg/ 100 g DW	mg/ 100 g DW	mg/ 100 g DW	mg/ 100 g FW	mg/ 100 g FW	mg/ 100 g	mg/ 100 g FW	mg/ 100 g DW	mg/ 100 g FW	mg/ 100 g DW	mg/ 100 g DW	mg/ 100 g DW	mg/ 100 g DW
Reference		[19]	[20]	[18]	[17]	[17]	[22]	[22]	[17]	[22]	[22]	[22]	[22]	[17]	[17]	[26]	[17]	[22]	[17]	[22]	[22]	[22]	[22]

DR- dry weight; FW- fresh weight.

**Table 3:** Vitamin content (per 100 g) of different varieties of almonds (*Prunus dulcis* Mill. D. A. Webb).

	Variety	Unknown	Unknown	Butte	Carmel	Fritz	Mission	Monterey	Nonpareil	Sonora	Units
Nutrients											
Thiamin		0.205	0.21								mg
Riboflavin		1.138	0.75	1.68	1.17	1.01	1.11	1	1.32	1.25	mg
Niacin		3.618	2.2	2.71	2.29	2.52	3.72	3.35	3.49	2.73	mg
Pantothenic acid		0.471									mg
Vitamin B6		0.137	0.15								mg
Total folate		44	49								μg
Vitamin E		25.63	24	27.6	29.9	26.3	28.3	21.9	26	31	mg
References		[19]	[20]	[17]							

**Table 4:** Sugar content of different varieties of almonds (*Prunus dulcis* Mill. D. A. Webb).

	Units	Sucrose				Raffinose		Glucose		Fructose		Maltose	Inositol
Almond Varieties		g/100 g FW	g/100 g DW	% DW	g/100 g FW	g/100 g DW	% DW	g/100 g DW	g/100 g DW	g/100 g DW	g/100 g DW	g/100 g DW	% DW
Butte					3.1								
Carmel					3.4								
Casanova		13.9				1.93		0.96		0.24			
Duro italiano		13.2				2.11		1.11		0.27			
Ferraduel		16.3				0.71		0.95		0.38			
Ferragnes		22.2		70.6–85.3		0.75	1.23–4.84	1.47		0.37			2.35–7.96
Ferrastar		22.0				1.73		1.04		0.17			
Fritz					3								
Gloriette		16.9				0.89		1.3		0.59			
Marcona		11.5				1.67		0.77		0.29			
Mission					2.9								
Monterey					3.7								
Nonpareil					4.1								
Pegarinhos (one seed)		12.0				1.43		0.71		0.19			
Pegarinhos (two seeds)		15.9				1.29		0.42		0.11			
Refego		14.7				1.4		0.68		0.25			
Sonora					3.1								
Sweet kernelled almond selections			2.53 (1.27–3.70)						1.88 (1.00–4.30)		4.08 (1.42–6.50)	0.92 (0.29–1.50)	
Bitter kernelled almond selections			2.52 (0.99–4.35)						2.24 (1.18–4.40)		2.98 (1.60–4.46)	0.79 (0.18–1.30)	
Reference		[27]	[28]	[23]	[17]	[27]	[23]	[27]	[28]	[27]	[28]	[28]	[23]

DR—dry weight; FW—fresh weight.

**Table 5:** Amino acid content of different varieties of almonds (*Prunus dulcis* Mill. D. A. Webb).

	Varieties	Almond	Carmel	Mission	Nonpareil
Amino Acids		(g/100 g protein)			
Histidine		0.539	1.93	1.93	2.14
Isoleucine		0.751	2.88	2.83	2.71
Leucine		1.473	6.19	5.77	5.81
Lysine		0.568	2.14	1.99	2.36
Methionine		0.157	0.4	0.37	0.42
Cystine		0.215	0.26	0.28	0.24
Threonine		0.601	1.95	1.91	1.93
Tryptophan		0.211	1.08	0.89	0.89
Valine		0.855	3.39	3.3	3.17
Phenylalanine		1.132	4.38	4.1	4.25
Tyrosine		0.45	1.51	1.39	1.19
Arginine		2.465	9.4	9.68	9.33
Alanine		0.999	4.25	4.12	4.11
Reference		[19]	[25]	[25]	[25]

**Table 6:** Clinical trials investigating the health-promoting properties of almonds.

Reference	Trial Type	Subjects	Number of Study Participants	Almond Intervention	Duration Intervention	Main Results
*Lipidemic control*						
[57]	R, CO	Normolipemic subjects	22	Replacement of 50% of usual daily intake of dietary fat with whole almonds or almond oil	6 weeks	Significantly reduced serum levels of triglycerides, TC and LDL-c and increased HDL-c
[58]	R, CO, C	Subjects with mild hypercholesterolemia	20	20 g almonds daily	6 weeks	Significantly reduced serum levels of TC, LDL-c and non-HDL-c
[59]	R, C	Healthy adults	85	56 g almonds daily	20 weeks	Significantly reduced TC, LDL-c, non-HDL-c, triglycerides, FM and WHR
[60]	R, OL, C	Subjects with hyperlipidemia	97	10 mL almond oil twice daily	4 weeks	Significantly reduce serum levels of TC and LDL-c
*Glycemic control*						
[61]	R, C	Healthy subjects	15	Test meal with 60 g almonds	-	Significantly reduced postprandial glycaemia, insulinemia and increased the concentration of serum protein thiol, indicating less oxidative protein damage
[62]	R, CO, C	Healthy subjects/T2DM patients	13/7	Test meal with 28 g almonds	-	Significantly reduced postprandial glycaemia in the T2DM group
		T2DM patients	13	1 serving (28 g) of almonds five days a week	12 weeks	Significantly reduced HbA1c in the T2DM group
[63]	R, C, PG	Young healthy adults	73	56.7 g almonds daily	8 weeks	A smaller decline in HDL-c; lower 2-h glucose AUC, IRI and higher Matsuda index during the OGTT, compared to control. Reduced fasting glucose and LDL-c
[65]	R, CO, C	T2DM patients with mild hyperlipidemia	20	Diet with almonds (20% of energy intake)	4 weeks	Significantly decreased levels of TC, LDL-c, LDL/HDL ratio, ApoB, ApoB/ApoA1 ratio, non-esterified fatty acid, fasting insulin, fasting glucose and HOMA-IR
*Obesity*						
[70]	R, C, PG	Overweight /obese subjects (BMI: 25‒40 kg/m^2^)	86	Hypocaloric, almond-enriched diet (15% energy from almond)	12 weeks	Greater reduction in truncal, visceral and total fat, diastolic blood pressure
[71]	R, CO	Obese adults	123	Hypocaloric, almond-enriched diet (28 g daily)	18 months	Less bodyweight reduction and no significant changes in body composition
*Cardiovascular risk*						
[64]	Non-C	Patients with T2DM	50	Diet with raw almond (20% of energy intake)	24 weeks	Significantly improved WC, waist-to-height ratio, TC, triglycerides, LDL-c, HbA1c, hs-CRP
[72]	R, CO, C	Subjects with elevated LDL-c	48	A cholesterol-lowering diet with 1.5 oz. almond/day	6 weeks	Significantly reduced non-HDL-c and LDL-c levels accompanied by reduced abdominal and leg fat
[73]	R, CO, C	Patients with CAD	45	Diet with 85 g almonds daily	6 weeks	Unchanged vascular outcomes (vascular function, peripheral arterial tonometry, pulse wave velocity), serum parameters (lipid, CRP, TNFα, E-selectin) and blood pressure. Reduced VCAM1 and increased urinary NO.
[74]	Non-C	Healthy men mild hyperlipidemia	30	60 g of almonds daily	4 weeks	Significantly decreased LDL-c, TC and ApoB100 levels
[75]	R, CO, C	Hyperlipidemic subjects	27	Isoenergetic supplements: full-dose almonds (73 g daily), half-dose almonds+half-dose muffins	4 weeks	Significantly reduced levels of LDL-c, LDL-c/HDL-c ratio, lipoprotein A and ox-LDL
[76]	R, CO, C	Hyperlipidemic subjects	27	Isoenergetic supplements: full-dose almonds (73 g daily), half-dose almonds+half-dose muffins	4 weeks	Increased content of OA and MUFA in TAG and NEFA fractions, which are inversely associated with both Framingham 10-year CHD risk score and CHD lipid risk
[77]	R, PG	Adults with prediabetes	65	ADA diet containing 20% of energy from almonds	16 weeks	Significantly reduced levels of insulin, HOMA-IR, HOMA-β, LDL-c
*Inflammation*						
[78]	R, CO, C	Healthy adults	25	Low-almond diet (10% isoenergetic replacement with almond) and high-almond diet (20% isoenergetic replacement with almonds)	4 weeks	Significantly reduced levels of E-selectin in high-almond diet and significantly reduced levels of CRP in both diets
*Oxidative stress*						
[79]	R, C	Healthy subjects, regular smokers	30	86 g and 164 g almonds daily	4 weeks	Significantly reduced levels of 8-OH-dG, MDA and single-strand DNA breaks. No significant effects on SOD and GSH-Px.
[80]	R, CO, C	Healthy subjects, regular smokers	60	84 g almonds daily	4 weeks	Significantly increased levels of SOD and GSH-Px and reduced levels of 8-OH-dG, MDA and DNA strand breaks
*Serum uric acid*						
[81]	R, C	CAD patients	150	10 g almonds	12 weeks	Significant reduced uric acid serum levels

*Abbreviations:* 8-OH-dG, 8-hydroxy2′-deoxyguanosine; ADA, American Diabetes Association; ApoB100, apolipoprotein B100; AUC, area under the curve; BMI, body mass index; C, controlled; CAD, coronary artery disease; CO, crossover; CRP, C-reactive protein; FM, fat mass; GSH-Px, glutathione peroxidase; HbA1c, glycosylated hemoglobin; HDL-c, high-density lipoprotein cholesterol; HOMA-IR, homeostatic model analysis for insulin resistance; HOMA-β, homeostatic model analysis for beta-cell function; hs-CRP, high-sensitivity C-reactive protein; IRI, insulin resistance index; LDL-c, low-density lipoprotein cholesterol; MDA, malondialdehyde; MUFA, monounsaturated fatty acid; NEFA, non-esterified fatty acid; NO, nitric oxide; non-C; non-controlled; OA, oleic acid; OGTT, oral glucose tolerance test; OL, open-label; oxLDL, oxidized LDL; PG, parallel-group; R, randomized; SOD, superoxide dismutase; T2DM, type 2 diabetes mellitus; TAG, triacylglycerides; TC, total cholesterol; TC, total cholesterol; TNFα, tumor necrosis factor-α; VCAM1, vascular cell adhesion molecule-1; WC, waist circumference; WHR, waist-to-hip ratio.

## Abbreviations

**AED**	almond-enriched hypocaloric diet;
**BMI**	body mass index;
**CHD**	coronary heart disease;
**CRP**	C reactive protein;
**GLP-1**	glucagon-like peptide-1;
**HbA1c**	glycosylated hemoglobin;
**GSH-Px**	glutathione peroxidase;
**HDL-c**	high-density lipoprotein cholesterol;
**HOMA-IR**	homeostatic model analysis for insulin resistance;
**HOMA-β**	homeostatic model analysis for beta-cell function;
**LDL-c**	low-density lipoprotein cholesterol;
**MDA**	malondialdehyde;
**MUFA**	monounsaturated fatty acids;
**NFD**	nut-free hypocaloric diet;
**PUFA**	polyunsaturated fatty acids;
**SOD**	superoxide dismutase;
**TC**	total cholesterol;
**TG**	triglycerides;
**TNF**	tumor-necrosis factor;
**T2DM**	type 2 diabetes mellitus;
**8-OH-dG**	8-hydroxy-2′-deoxyguanosine.
